# Relationships between received and perceived social support and health-related quality of life among patients receiving methadone maintenance treatment in Mainland China

**DOI:** 10.1186/s13011-017-0116-3

**Published:** 2017-06-26

**Authors:** Kaina Zhou, Hengxin Li, Xiaoli Wei, Juan Yin, Peifeng Liang, Hongmei Zhang, Lingling Kou, Mengmeng Hao, Lijuan You, Xiaomei Li, Guihua Zhuang

**Affiliations:** 1 Xi’an Jiaotong University Health Science Center, No. 76 Yanta West Road, Xi’an, Shaanxi 710061 China; 2 Xi’an Center for Disease Control and Prevention, No. 599 Xiying Road, Xi’an, Shaanxi 710054 China

**Keywords:** Social support, Health-related quality of life, Methadone maintenance treatment

## Abstract

**Background:**

Social support has been considered one of the most important factors of health-related quality of life (HRQoL) evaluations among different populations; however, few studies have explored the relationships of both received and perceived social support to HRQoL among patients undergoing methadone maintenance treatment (MMT). Thus, the purpose of this cross-sectional study was to clarify these relationships.

**Methods:**

Participants were patients admitted at the two largest privately and publicly funded MMT clinics in Xi’an. The main explanatory variable was social support, both received (i.e., social network support and professional support services) and perceived (Multidimensional Scale of Perceived Social Support). The outcome was HRQoL, which was evaluated using the Short-Form 36 Health Survey version 2 (SF-36v2) and the Quality of Life Scale for Drug Addicts (QOL-DAv2.0). We carried out independent samples *t*-tests and multiple linear regression analysis to examine the relationships between received and perceived social support and HRQoL.

**Results:**

The study findings revealed that patients with good social support had significantly higher scores on the SF-36v2 and QOL-DAv2.0 (*p* < 0.05). After controlling for individual characteristics, the significant factors predicting HRQoL were good family relationships, usually communicating with others, a convenient service time, appropriate treatment charges, and good perceived social support (*p* < 0.05).

**Conclusions:**

Our results suggest that received and perceived social support influences HRQoL among individuals receiving MMT. Thus, these variables should be considered during health management efforts and interventions directed at this patient population.

## Background

Health-related quality of life (HRQoL) is a multifactorial construct describing individuals’ perceptions of their own physical, psychological, and social functioning [1]. It has been regarded as a valuable patient-reported outcome in evaluations of therapeutic effectiveness and health for patients undergoing methadone maintenance treatment (MMT) [2]. MMT is currently the most effective therapeutic approach for opioid dependence. China initiated MMT in 2004 (with 8 clinics serving 1029 drug users); by 2012, a total of 748 clinics were offering MMT to over 360,000 drug users [3]. The patient pays 10 RMB per visit to the clinician and receives methadone under staff supervision, in both publicly and privately funded clinics. Patients with HIV can receive MMT for free, in an effort to stem the spread of the disease through sharing of infected needles. In comparison to the general population, MMT patients have poorer physical and psychological health and show considerable impairments in social functioning [4–6]. Numerous published studies have explored the determinants of HRQoL [7–9], among which the most important may be social support [10–12].

A previous literature review demonstrated that social support comprises both structural and functional components [13], with the former being composed of formal and informal support (e.g., the size of an individual’s social network, the frequency of contact with network members, presence of reciprocal support and quality of such support) and the latter the perceived level of support received (e.g., emotional and tangible support). These two components can be broadly distinguished as “received” (i.e., objective) and “perceived” (i.e., subjective) support, and both are important for an individual’s well-being [14].

Social support has been found to be an important factor relating to HRQoL among different populations, such as patients with heart failure [15], diabetes [16], coronary heart disease [17], Hodgkin’s lymphoma [18], polyneuropathy [19], patients undergoing hemodialysis [20], cancer [21, 22], earthquake survivors [23], caregivers [24], HIV/AIDS [25], and substance dependence [26]. Despite this plethora of research, these past studies only considered one aspect of social support—either received or perceived—thus preventing a more comprehensive understanding of the relationships between social support and HRQoL. Similar effects of social support have been found among MMT patients: namely, Lin et al. [27] reported that perceived family support was associated with increased physical, psychological, and social functioning in this population. However, this previous study did not consider perceived support from friends or significant others. Furthermore, apart from perceived social support, MMT patients often receive actual support from their personal social network (e.g., family, friends) and clinical services (e.g., professional support) [28]; however, despite this, few studies have focused on the relationships between such objective forms of social support and HRQoL among patients receiving MMT.

To clarify this issue, we comprehensively explored the relationships between objective (i.e., received social network support and professional support services) and subjective (i.e., perceived) forms of social support and HRQoL, among MMT patients from Mainland China. Our hypothesis was that both received and perceived social support would correlate positively with HRQoL. To our knowledge, this is the first study considering both objective and perceived social support in a same sample of MMT patients. The study findings will help inform health management efforts and intervention programs targeting the MMT patient population.

## Methods

### Design

This was a cross-sectional design examining the relationships between received and perceived social support and HRQoL, among patients receiving MMT.

### Subjects and data collection

Participants were patients who had been admitted to one of the two largest MMT clinics (i.e., the publicly funded Xinan Clinic and the privately funded Minle Clinic) in Xi’an, China. The inclusion criteria were being at least 18 years, providing written informed consent, and being fluent in Chinese. Exclusion criterion were patients with cognitive disorders.

The collected data broadly included individual characteristics (14 items, e.g., age, gender, education level, marital status, employment status), social network support (3 items), professional support services (5 items), perceived social support, and HRQoL. Individual, face-to-face interviews, administered by trained interviewers in a quiet and well-lit room, were used to collect data from recruited participants.

### Measurements

#### Social support

Social support was the main explanatory variable, and was measured in terms of received and perceived social support.

#### Received social support

According to the definition of social support [13], received social support was evaluated in terms of social network support and professional support services, which reflect support from patients’ social network and MMT clinical staff, respectively. Social network support was assessed using 3 items (i.e., good family relationships, usually communicating with others, and having family/friends who support MMT), while professional support services were assessed using 5 items (i.e., having a convenient service time, having a good service attitude, usually receiving health counseling, usually receiving psychological counseling, and appropriate treatment charges). These 8 items were developed, under the direction of two MMT specialists, with reference to the National Community Methadone Maintenance Treatment Assessment Questionnaire, a standardized questionnaire used in MMT clinics throughout Mainland China. Participants answered each item by indicating whether they had received the corresponding form of social network support or professional support service (i.e., “yes” or “no”). We used answers to the individual items of received social support in the analysis instead of a summed total score.

#### Perceived social support

Perceived social support was assessed using the Multidimensional Scale of Perceived Social Support (MSPSS) [29]. Each item is rated on a 7-point Likert scale, with a total score range from 12 to 84. A total score of 50 or more represents good perceived social support. Published data have shown that the MSPSS has high internal consistency reliability (Cronbach’s α: 0.92) and excellent factorial validity [30–32].

#### HRQoL

HRQoL was the outcome variable and was measured using two instruments: a generic instrument, the Short-Form 36 Health Survey version 2 (SF-36v2), with a physical component summary (PCS) and a mental component summary (MCS) [33–35], and a more specific instrument, the Quality of Life Scale for Drug Addicts (QOL-DAv2.0) [35–37]. The items of the QOL-DAv2.0 are rated on a 5-point Likert scale, and the total score ranges from 40 to 200. We used both measures to obtain a more comprehensive assessment of HRQoL among MMT patients.

### Data analyses

A database was constructed using EpiData 3.1, and all data were double-entered by two data managers to capture any possible data entry errors. Frequencies and percentages were used to describe categorical variables, while means and standard deviations were used to describe continuous variables. Independent samples *t*-tests were performed to compare HRQoL according to each form of social support (i.e., received or perceived). Multiple linear regression analysis was employed to identify the factors influencing HRQoL after controlling for individual characteristics (i.e., age, gender, education level, marital status, having children, employment status, average monthly income over the past year, stable income, living with family, chronic disease, initial drug use age, drug use over the past month, days of receiving MMT, and average daily methadone dose); categorical variables were entered as dummy variables. The SF-36v2PCS, SF-36v2MCS, and QOL-DAv2.0 were regarded as the dependent variables, respectively. All statistical analyses were completed using IBM SPSS Statistics 22.0 (IBM Corp., Armonk, NY). A *p* < 0.05 (two-tailed) was considered to be statistically significant.

### Ethical approval

The study protocol was reviewed and approved by the Human Research Ethics Committee of Xi’an Jiaotong University. Written informed consent was obtained from each participant before the questionnaire was administered.

## Results

Of the 1270 patients eligible for the study, 1212 completed the questionnaire survey, with 361 (29.8%) in the Xinan clinic and 851 (70.2%) in the Minle clinic. The 58 (4.6%) participants (30 in the Xinan clinic and 28 in the Minle clinic) who were eligible, but did not complete the questionnaire, refused to provide written informed consent. All patients had self-paid for their MMT, except for those of positive HIV serostatus. Regarding the questionnaire, all patients understood the questions well and completed the questionnaire in its entirety. Each interview took 20–25 min. Individual characteristics and social support levels are shown in Table 1. Regarding the total score of perceived social support, around 66.7% of participants exhibited good perceived social support (*n* = 809; Table 1), according to the cutoff score of 50 [31].

**Table 1:** Individual characteristics and social support (*N* = 1212) (*n*, %)

Individual characteristics and social support	*n* (%)
Individual characteristics	
Age (years) (mean ± SD) (range: 21–65)	42.48 ± 6.24
Gender	
Male	934 (77.1)
Female	278 (22.9)
Education level	
Primary or below	141 (11.6)
Secondary	987 (81.4)
Tertiary	84 (6.9)
Marital status	
Married	705 (58.2)
Others	507 (41.8)
Having children	
Yes	840 (69.3)
No	372 (30.7)
Employment status	
Unemployed	594 (49.0)
Employed	618 (51.0)
Average monthly income over the past year (Chinese yuan)	
< 1000	708 (58.4)
1000–3000	307 (25.3)
> 3000	197 (16.3)
Stable income	
Yes	356 (29.4)
No	856 (70.6)
Living with family	
Yes	898 (74.1)
No	314 (25.9)
Chronic disease	
Yes	616 (50.8)
No	596 (49.2)
Initial drug use age (years) (mean ± SD) (range: 14–52)	28.41 ± 7.35
Drug use over the past month	
Yes	237 (19.6)
No	975 (80.4)
Days of receiving MMT (mean ± SD) (range: 1–1659)	717.15 ± 435.57
Average daily methadone dose (mg) (mean ± SD) (range: 8–106)	49.60 ± 15.42
Social network support	
Good family relationships	
Yes	820 (67.7)
No	392 (32.3)
Usually communicates with others	
Yes	455 (37.5)
No	757 (62.5)
Has family/friends who support MMT	
Yes	1065 (87.9)
No	147 (12.1)
Professional support services	
Convenient service time	
Yes	1069 (88.2)
No	143 (11.8)
Good service attitude	
Yes	1062 (87.6)
No	150 (12.4)
Usually receives health counseling	
Yes	891 (73.5)
No	321 (26.5)
Usually receives psychological counseling	
Yes	597 (49.3)
No	615 (50.7)
Appropriate treatment charges	
Yes	793 (65.4)
No	419 (34.6)
Perceived social support	
Perceived support from family (mean ± SD)	19.48 ± 4.65
Perceived support from friends (mean ± SD)	17.42 ± 5.08
Perceived support from significant others (mean ± SD)	18.16 ± 4.60
Global perceived social support (mean ± SD)	55.06 ± 12.40
Good perceived social support ^a^	
Yes	809 (66.7)
No	403 (33.3)

^a^Good perceived social support (yes or no) was divided according to the cutoff of 50

*MMT* methadone maintenance treatment. *SD* standard deviation

Regarding the SF-36v2, the mean scores of the summary components were 48.62 ± 7.94 (PCS) and 41.02 ± 10.74 (MCS), while the mean scores of the eight subscales were ranked as follows: 49.83 ± 7.39 (physical functioning), 48.24 ± 10.86 (bodily pain), 46.93 ± 10.43 (vitality), 45.43 ± 10.48 (role-physical), 44.52 ± 10.07 (social functioning), 41.66 ± 10.48 (mental health), 41.56 ± 12.01 (role-emotional), and 39.74 ± 11.02 (general health). All these scores were lower than the norm of 50 [34]. The mean total score of the QOL-DAv2.0 was 64.45 ± 17.48, while the scores of the four subscales were 75.43 ± 20.61 (symptoms), 67.61 ± 24.01 (psychology), 56.99 ± 19.64 (physiology), and 56.97 ± 17.26 (society).

Patients with good family relationships, who usually communicated with others, and who had a convenient service time, appropriate treatment charge, and good perceived social support displayed higher SF-36v2 and QOL-DAv2.0 scores than did those without good social support (*p* < 0.05). Furthermore, participants reporting good service attitude had higher scores on the PCS [mean difference: 2.30, 95% confidence interval: (0.95, 3.66), *p* = 0.001] and QOL-DAv2.0 [4.31 (1.32, 7.29), *p* = 0.002], while patients who usually received health or psychological counseling had higher scores on the MCS [2.00 (0.63, 3.36), *p* = 0.004; 1.35 (0.14, 2.56), *p* = 0.029] and QOL-DAv2.0 [2.88 (0.65, 5.11), *p* = 0.011; 2.42 (0.45, 4.39), *p* = 0.016; Table 2].

**Table 2:** Mean difference (MD) in health-related quality of life by social support status (independent samples *t*-test; *N* = 1212)

Social support	SF-36v2PCS		SF-36v2MCS		QOL-DAv2.0	
	MD (95% CI)	*P*	MD (95% CI)	*P*	MD (95% CI)	*P*
Received social support						
Social network support						
Good family relationship (yes vs. no)	3.82 (2.89, 4.75)	<0.001	6.85 (5.61, 8.08)	<0.001	14.82 (12.88, 16.75)	<0.001
Usually communicates with others (yes vs. no)	3.68 (2.77, 4.58)	<0.001	6.45 (5.25, 7.64)	<0.001	12.11 (10.20, 14.03)	<0.001
Has family/friends who support MMT (yes vs. no)	0.28 (−1.10, 1.65)	0.69	0.84 (−1.01, 2.70)	0.84	1.36 (−1.65, 4.38)	0.38
Professional support services						
Convenient service time (yes vs. no)	2.40 (1.02, 3.79)	0.001	3.16 (1.29, 5.03)	0.001	5.61 (2.57, 8.65)	<0.001
Good service attitude (yes vs. no)	2.30 (0.95, 3.66)	0.001	1.62 (−0.22, 3.46)	0.08	4.31 (1.32, 7.29)	0.002
Usually receives health counseling (yes vs. no)	0.93 (−0.09, 1.94)	0.07	2.00 (0.63, 3.36)	0.004	2.88 (0.65, 5.11)	0.011
Usually receives psychological counseling (yes vs. no)	0.65 (−0.25, 1.54)	0.16	1.35 (0.14, 2.56)	0.029	2.42 (0.45, 4.39)	0.016
Appropriate treatment charges (yes vs. no)	3.32 (2.40, 4.24)	<0.001	4.16 (2.91, 5.41)	<0.001	7.43 (5.40, 9.46)	<0.001
Perceived social support						
Good perceived social support (yes vs. no)	3.14 (2.21, 4.08)	<0.001	6.55 (5.31, 7.78)	<0.001	11.86 (9.88, 13.84)	<0.001

*SF-36v2* Short-Form 36 Health Survey Version 2

*PCS* physical component summary

*MCS* mental component summary

*QOL-DAv2.0* Quality of Life Scale for Drug Addicts

*MMT* methadone maintenance treatment

*95% CI* 95% confidence interval

The correlation coefficients among perceived social support and received social support ranged from 0.03 to 0.21. In the multiple regression analysis, after controlling for individual characteristics, the significant factors affecting HRQoL were not having good family relationships, not usually communicating with others, not having a convenient service time, not having appropriate treatment charges, and not having good perceived social support (*p* < 0.05; Table 3).

**Table 3:** Effects of social support on health-related quality of life (multiple linear regression analysis^a^; *N* = 1212)

Independent variable	SF-36v2PCS		SF-36v2MCS		QOL-DAv2.0	
	B (95%CI)	*P*	B (95%CI)	*P*	B (95%CI)	*P*
Received social support						
Social network support						
Good family relationship (yes vs. no)	−1.80 (−2.77, −0.84)	<0.001	−3.87 (−5.19, −2.55)	<0.001	−9.22 (−11.22, −7.22)	<0.001
Usually communicating with others (yes vs. no)	−1.00 (−1.94, −0.06)	0.038	−2.79 (−4.08, −1.49)	<0.001	−4.19 (−6.15, −2.23)	<0.001
Family/friends supporting MMT (yes vs. no)	0.44 (−0.80, 1.68)	0.49	0.53 (−1.17, 2.22)	0.55	1.27 (−1.30, 3.84)	0.33
Professional support services						
Convenient service time (yes vs. no)	−2.17 (−3.44, −0.91)	0.001	−2.20 (−3.94, −0.47)	0.013	−4.44 (−7.07, −1.81)	0.001
Good service attitude (yes vs. no)	−1.11 (−2.42, 0.20)	0.10	0.42 (−1.38, 2.22)	0.65	−0.97 (−3.69, 1.76)	0.49
Usually receiving health counseling (yes vs. no)	0.27 (−0.81, 1.35)	0.63	−0.56 (−2.05, 0.92)	0.46	0.47 (−1.78, 2.71)	0.68
Usually receiving psychological counseling (yes vs. no)	−0.05 (−0.99, 0.89)	0.92	−0.35 (−1.64, 0.95)	0.87	−1.07 (−3.02, 0.89)	0.28
Appropriate treatment charge (yes vs. no)	−1.22 (−2.10, −0.33)	0.007	−1.92 (−3.13, −0.70)	0.002	−2.70 (−4.53, −0.87)	0.004
Perceived social support						
Good perceived social support (yes vs. no)	−1.47 (−2.35, −0.59)	0.001	−4.23 (−5.44, −3.02)	<0.001	−7.09 (−8.91, −5.26)	<0.001

^a^Multiple linear regression analysis was performed after controlling for the following dummy variables: gender (ref. male), education level (ref. primary and below), marital status (ref. married), having children (ref, yes), employment status (ref. unemployed), average monthly income over the past year (Chinese yuan, ref. <1000), stable income sources (ref. yes), living with family (ref. yes), chronic disease (ref. yes), and drug use over the past month (ref. yes), as well as the continuous characteristics (age, initial drug use age, days of receiving MMT, and average daily methadone dose)

*SF-36v2* Short-Form 36 Health Survey Version 2

*PCS* physical component summary

*MCS* mental component summary

*QOL-DAv2.0* Quality of Life Scale for Drug Addicts

*MMT* methadone maintenance treatment

*95% CI* 95% confidence interval

## Discussion

The findings overall supported our hypothesis that both objective and perceived social support positively correlate with HRQoL. In other words, MMT patients with good objective and perceived social support tended to have better HRQoL. These results demonstrate the key roles of good social support in HRQoL enhancement in an MMT patient population.

We found that MMT patients had poorer overall mental health than physical health, and demonstrated specific impairments in general health among the eight subscales of the SF-36v2. Notably, the scores of the two summary components and eight subscales were all lower than the norm of 50 [34], demonstrating that MMT patients have poorer HRQoL than do the general population, especially in terms of overall mental and general health [38]. The relatively lower scores on the social, physical, and psychological function subscales of the QOL-DAv2.0 indicated that impairments to these domains of health are common among MMT patients. This is consistent with the results of a study by Zhang et al. [37], which demonstrated that MMT patients have poor HRQoL, especially in terms of physical and social functioning. Therefore, further efforts should be taken to improve physical, psychological, and social health among MMT patients during their treatment, in addition to targeting other, more objective therapeutic outcomes.

Having good family relationships and usually communicating with others were significant social network support factors that influenced HRQoL. Having good family relationships is important for ensuring that these individuals acquire care and support from their family, while usually communicating with others can be said to reflect participants’ physical and mental health status, especially from the perspective of social functioning [6]. In this study, however, 32.3% of the patients did not have a good family relationship and 62.5% did not usually communicate with others, suggesting that these patients lacked an adequate environment for social communication with their family, friends, or significant others. This may have been due to stigmatization, discrimination, and/or misunderstanding of MMT [27, 28]. Notably, unlike having a good family relationship or communicating with others, having family/friends who supported MMT did not significantly relate to HRQoL in this study. Therefore, interventions, such as MMT-related health education, might be provided to patients’ family members, friends, and significant others in order to reduce their negative attitudes and establish a good communication environment for the patients.

Regarding professional support services, we found that only a convenient service time and appropriate treatment charges were significantly related to HRQoL. Both factors have been considered to be strong protective predictors of treatment adherence among MMT patients [39–41], which may be beneficial to their HRQoL. Therefore, MMT clinical staff should attempt to adjust service times and treatment charges to suit patients’ needs, which in turn could help improve these outcomes. Unlike social network support, professional support services are mainly provided by clinical staff within a treatment context. Other support services, such as service attitude, and health and psychological counseling, were also helpful for HRQoL improvement. However, after controlling for individual characteristics, these factors were no longer significantly related to HRQoL. This difference is likely due to clinical staff’s negative views toward MMT patients or deficiencies in complementary clinical services. Therefore, efforts should be made to further improve professional support services. Programs such as service provider training, home visits combined with telephone calls, and contingency management interventions are worth considering [42, 43].

Perceived social support had a significant effect on HRQoL. For MMT patients, family support is perhaps the most pivotal aspect of their social support system [28, 29]. MMT patients who perceive themselves as having good family support are also likely to perceive themselves as having a physically secure environment, adequate health and social care, financial support, the ability to utilize social resources, and a beneficial home environment [6, 44], all of which can improve HRQoL. Accordingly, family-focused intervention programs may be needed to improve the degree of perceived family support in MMT patients [27]. Considering that friends and significant others are also components of good perceived social support, further efforts should also be made to reduce negative opinions, misunderstanding, stigmatization, and discrimination, which may, in turn, improve MMT patients’ perceptions of support from their friends and significant others [27, 28].

The findings of the current study have shown that good social network support, professional support services, and perceived social support are all correlated with HRQoL, and that patients with good perceived social support demonstrated higher scores on the SF-36v2 and the QOL-DAv2.0, when compared to individuals without good perceived social support. These findings also support the idea that both good HRQoL and social support reflect good recovery capitol for patients with MMT, which is beneficial to patients during long-term rehabilitation. Furthermore, the findings suggest that, aside from routine treatments, MMT clinical staff might offer more comprehensive interventions for patients and their family members, friends, and/or significant others, such as health education or counseling, family support training, and MMT knowledge lectures, all of which can be useful for establishing an accurate understanding of MMT among patients and reducing negative attitudes toward MMT patients among family members, and perhaps, society at large. Yang et al. [45] concluded that dissemination of more accurate MMT knowledge and resolution of conflicting perspectives of MMT are urgently needed to increase societal support for MMT patients. With sufficient support received from society, patients’ perceived support would likely demonstrate a corresponding increase, thereby helping improve their HRQoL during long-term MMT.

Although we observed positive relationships between received and perceived social support and HRQoL in this MMT patient sample from Mainland China, it remains unclear whether this relationship would exist in the MMT patient populations of other countries. Indeed, given the deficiency of investigations on the differences in social support relationships with HRQoL between cultures, regardless of the patient population, it would be worthwhile to further consider the influence of culture on these relationships.

This study has some limitations. First, unobserved factors were not considered; as a result, the findings may be subject to possible confounding factors. Second, although the results from this study revealed possible relationships between social support and HRQoL, these relationships should not be interpreted as causal. Third, we did not collect any information on other infectious diseases. Finally, this study was conducted only in Xi’an, China, thereby limiting its generalizability.

## Conclusions

Based on the study findings, MMT patients had relatively poor HRQoL, which is influenced by both objective and perceived social support. The positive correlation between good social support and good HRQoL also reflects good recovery capitol for patients with MMT during long-term rehabilitation. It is recommended that social support be considered in health management and intervention programs targeting the MMT patient population.

## Abbreviations

HRQoL: Health-related quality of life
MCS: Mental component summary
MMT: Methadone maintenance treatment
MSPSS: Multidimensional Scale of Perceived Social Support
PCS: Physical component summary
QOL-DAv2.0: Quality of Life Scale for Drug Addicts
SF-36v2: Short-Form 36 Health Survey version 2
